# Editorial: Dendritic Cell and Macrophage Nomenclature and Classification

**DOI:** 10.3389/fimmu.2016.00168

**Published:** 2016-05-02

**Authors:** Florent Ginhoux, Martin Guilliams, Shalin H. Naik

**Affiliations:** ^1^Singapore Immunology Network (SIgN), A*STAR, Singapore; ^2^Laboratory of Immunoregulation, VIB Inflammation Research Center, Ghent University, Ghent, Belgium; ^3^Department of Respiratory Medicine, Ghent University, Ghent, Belgium; ^4^Molecular Medicine Division, The Walter + Eliza Hall Institute of Medical Research, The University of Melbourne, Melbourne, VIC, Australia; ^5^Department of Medical Biology, The University of Melbourne, Melbourne, VIC, Australia

**Keywords:** dendritic cells, macrophages, monocytes, nomenclature

**The Editorial on the Research Topic**

**Dendritic Cell and Macrophage nomenclature and classification**

Mononuclear phagocytes that include dendritic cells (DCs), monocytes, and macrophages constitute a group of cell types crucial for the control of pathogens and induction of immune responses as well as for the support of tissue functions. These properties make them highly relevant targets for immune therapy, vaccination, and treatment of autoimmune and inflammatory diseases (1, 2). However, exactly how many cell types exist in the mononuclear phagocyte system (MPS), or whether they even combine to constitute a family, has been a matter of contention for many years. Historically, cells of the MPS have, at one time or another, been referred to as erythrophagocytes, pyrrhol cells, adventitia cells, rhagiocrine cells, polyblasts, clasmatocytes, and histiocytes (Yona and Gordon) prior to their current terminology established in 1972 (3). The seminal discovery of a new cell type termed DCs in the 1970s by the late Ralph Steinman that were distinct from macrophages added even more complexity in the MPS classification (4). However, some time passed before DCs were fully accepted as true member of the MPS. Over time, there was appreciation that there were not just one but multiple DC subtypes, each with a specialized role (5). So, while a “*dendritic-shaped cell that can process and present antigen to activate naive T cells*” was a good initial working definition (6), it did not take into account the inconsistent observations that other cells can be dendritic in appearance or activate naive T cells, and that not all “DCs” are immunostimulatory nor dendritic (7). As a result, many different cell types have been given a DC moniker over the years, such as monocyte-derived DCs, conventional DCs (cDCs), and plasmacytoid DCs (8). This appreciation of multiple subtypes has both clarified and confused the field. Importantly, we do not consider the classification and nomenclature issues as trivial semantics. Indeed, classification is of very practical importance in allowing researchers to work to a common framework as highlighted by Norma Lang “If we cannot name it, we cannot control it, finance it, teach it, research it, or put into public policy (page 109)” (9).

The idea behind this Frontiers Research Topic on “Dendritic Cell and Macrophage Nomenclature and Classification” was to have an open debate on the advantages and disadvantages of different classification systems of cells within the MPS. In this Research Topic, 17 contributions from international experts cover the complexity of the MPS, from its ontogeny and transcriptional regulation, its classification in different tissues and different species.

First, in a historical perspective, Yona and Gordon examine the early origins and development of macrophage research from Ilya Metchnikoff’s discovery to the establishment of the MPS nomenclature half a century ago.

In an opinion article, Vremec and Shortman discuss issues of DC subset definition encountered in their past work.

In a hypothesis and theory article, Guilliams and van de Laar discuss the practical application of our recently proposed classification system based on ontogeny (8).

Hoeffel and Ginhoux cover the ontogeny of tissue-resident macrophages and discuss evidence suggesting that hematopoietic stem cell-independent embryonic precursors transiently present in the yolk sac and the fetal liver give rise to long-lasting self-renewing macrophage populations.

Tussiwand and Gautier discuss the developmental pathways of murine MPS cells, with a particular emphasis on the transcriptional factors that regulate their development and function.

Poltorak and Schraml review experimental approaches taken to fate map DCs and discuss how these have shaped our understanding of DC ontogeny and lineage affiliation.

In a perspective article, Gottschalk and Kurts review the complexity of the renal MPS, and how to distinguish DCs and macrophages in the kidney from the nephrologist’s point of view.

Gross et al. discuss origins and functions of intestinal DCs and macrophages and their respective subsets, focusing largely on the mouse and cells residing in the lamina propria.

Greter et al. discuss myeloid cells in the brain and the difficulties to delineate resident microglia from infiltrating myeloid cells using currently known markers and the recent advances that have helped to make clear definitions between phenotypically similar, yet functionally diverse myeloid cell types of the brain.

Cassado et al. review the heterogeneity of peritoneal macrophages, which exhibit distinct phenotypes, functions, and origins.

Eckert et al. summarize the multiple roles of macrophages and DCs in chronic liver diseases and outline the currently known marker combinations for the identification of these cell populations for the study of their role in liver immunology.

Moving to human cells, Reynolds and Haniffa review the parallel organization of human and mouse mononuclear phagocyte networks. They also discuss the strategies, power, and utility of comparative biology approaches to integrate recent advances in human and mouse mononuclear phagocyte biology, and its potential to drive forward clinical translation of this knowledge.

In a research article, Vu-Manh et al. extend our knowledge of the homology of the MPS across species through comparative transcriptomics. They present an approach combining refined cell sorting and integrated comparative transcriptomics analyses, which revealed conservation of the mononuclear phagocyte organization across human, mouse, sheep, pigs, and chicken.

In a complementary review, Vu-Manh et al. discuss the highly significant conservation during evolution of DC subsets cell surface phenotyping, expression analysis of hallmark genes, and functions.

Ziegler-Heitbrock reviews human blood monocyte heterogeneity and their subdivision into classical, intermediate, and non-classical monocytes, and how these proportions change during inflammation and discuss its relevance to management of disease.

Durand and Segura review recent advances in our understanding of the human DC network and discuss some remaining gaps and future challenges of the human DC field.

Finally, in an original research article, Sudan et al. identify novel markers of activated human macrophages through the analysis of gene-expression profiles for human macrophages of a single donor subjected to 33 distinct activating conditions.

Altogether, the many contributions to this Frontiers Research Topic not only underline the complexity of the MPS system but also highlight the similarities between MPS cells of different tissues. Moreover, classifying MPS cells based on their gene-expression profiles results in a classification system that is close to the classification of cells based on their cellular origin and development. Although a final basis for MPS classification has not been defined yet, we hope this Frontiers Research Topic will pave the way toward a wider consensus within the field.

## Conflict of Interest Statement

The authors declare that the research was conducted in the absence of any commercial or financial relationships that could be construed as a potential conflict of interest.

## References

[1] Ginhoux F Jung S. Monocytes and macrophages: developmental pathways and tissue homeostasis. Nat Rev Immunol (2014) 14:392–404. 10.1038/nri3671 24854589

[2] Schlitzer A Ginhoux F. Organization of the mouse and human DC network. Curr Opin Immunol (2014) 26:90–9. 10.1016/j.coi.2013.11.002 24556405

[3] van Furth R Cohn ZA Hirsch JG Humphrey JH Spector WG Langevoort HL. The mononuclear phagocyte system: a new classification of macrophages, monocytes, and their precursor cells. Bull World Health Organ (1972) 46:845–52. 4538544 PMC2480884

[4] Steinman RM Cohn ZA. Identification of a novel cell type in peripheral lymphoid organs of mice. I. Morphology, quantitation, tissue distribution. J Exp Med (1973) 137:1142–62. 10.1084/jem.137.5.1142 4573839 PMC2139237

[5] Schlitzer A McGovern N Ginhoux F. Dendritic cells and monocyte-derived cells: two complementary and integrated functional systems. Semin Cell Dev Biol (2015) 41:9–22. 10.1016/j.semcdb.2015.03.011 25957517

[6] Naik SH. Demystifying the development of dendritic cell subtypes, a little. Immunol Cell Biol (2008) 86:439–52. 10.1038/icb.2008.28 18414430

[7] Perié L Naik SH Toward defining a “lineage” – the case for dendritic cells. Semin Cell Dev Biol (2015) 41:3–8. 10.1016/j.semcdb.2015.02.004 25701440

[8] Guilliams M Ginhoux F Jakubzick C Naik SH Onai N Schraml BU Dendritic cells, monocytes and macrophages: a unified nomenclature based on ontogeny. Nat Rev Immunol (2014) 14:571–8. 10.1038/nri3712 25033907 PMC4638219

[9] Clark J Lang N Nursing’s next advance: an internal classification for nursing practice. Int Nurs Rev (1992) 39:109–28. 1517047

